# Editorial

**Published:** 1982

**Authors:** 


### Editorial

### Progress Report

We were able to report recently that *Smith, Kline & French Laboratories Ltd.* had come to our aid with a donation sufficient to cover two numbers of this Journal. Another great Drug Firm, *Farmitalia Carlo Erba Ltd.* has made a similar donation which we now gratefully acknowledge. This company is a wholly owned subsidiary of Montedison, Italy's largest chemical company. They make a wide range of drugs but have recently come to the fore in the field of Cancer Chemotherapy with the discovery of *Adriamycin* which is active alone and in combination with other cytotoxic agents against a large number of malignancies. It is heartening to know that the survival of our Journal is a cause which these pharmaceutical giants are willing to support so handsomely.

Our readers will be aware that since the appearance of the last number of volume 95, there has been a long gap. This is not due to a shortage of material but to a 'logjam' at the printers. This problem bedevilled the previous editorship and led to two changes of printer in 5 years. We are assured that this time it is due to special circumstances which will not recur and that there can now be a quick succession of the numbers required to bring us up to date in January 1983 with the publication of our Centenary number. Meanwhile we must beg the forbearance of our readers and of our 'Contemporaries' with whom we exchange our Journal.

